# Percutaneous left stellate ganglion block for refractory ventricular tachycardia in structural heart disease: our single-centre experience

**DOI:** 10.1007/s12471-024-01880-w

**Published:** 2024-06-12

**Authors:** Vincent R. van der Pas, Jurren M. van Opstal, Marcoen F. Scholten, Nelson P. Monteiro de Oliveira, Ron G. H. Speekenbrink, Pascal F. H. M. van Dessel

**Affiliations:** 1 Department of Cardiology, Thorax Centrum Twente, Medisch Spectrum Twente, Enschede, The Netherlands; 2 Department of Anaesthesiology, Medisch Spectrum Twente, Enschede, The Netherlands; 3 Department of Cardiothoracic Surgery, Thorax Centrum Twente, Medisch Spectrum Twente, Enschede, The Netherlands

**Keywords:** Autonomic modulation, Sympathetic denervation, Stellate ganglion, Ventricular tachycardia

## Abstract

**Introduction:**

When electrical storm (ES) is amenable to neither antiarrhythmic drugs, nor deep sedation or catheter ablation, autonomic modulation may be considered. We report our experience with percutaneous left stellate ganglion block (PSGB) to temporarily suppress refractory ventricular arrhythmia (VA) in patients with structural heart disease.

**Methods:**

A retrospective analysis was performed at our institution of patients with structural heart disease and an implantable cardioverter defibrillator (ICD) who had undergone PSGB for refractory VA between January 2018 and October 2021. The number of times antitachycardia pacing (ATP) was delivered and the number of ICD shocks/external cardioversions performed in the week before and after PSGB were evaluated. Charts were checked for potential complications.

**Results:**

Twelve patients were identified who underwent a combined total of 15 PSGB and 5 surgical left cardiac sympathetic denervation procedures. Mean age was 73 ± 5.8 years and all patients were male. Nine of 12 (75%) had ischaemic cardiomyopathy, with the remainder having non-ischaemic dilated cardiomyopathy. Mean left ventricular ejection fraction was 35% (± 12.2%). Eight of 12 (66.7%) patients were already being treated with both amiodarone and beta-blockers. The reduction in ATP did not reach statistical significance (*p* = 0.066); however, ICD shocks (*p* = 0.028) and ATP/shocks combined were significantly reduced (*p* = 0.04). At our follow-up electrophysiology meetings PSGB was deemed ineffective in 4 of 12 patients (33%). Temporary anisocoria was seen in 2 of 12 (17%) patients, and temporary hypotension and hoarseness were reported in a single patient.

**Discussion:**

In this limited series, PSGB showed promise as a method for temporarily stabilising refractory VA and ES in a cohort of male patients with structural heart disease. The side effects observed were mild and temporary.

## What’s new?

- A percutaneous block of the left stellate ganglion appeared effective in reducing the ventricular arrhythmia burden in patients with refractory ventricular arrhythmia in the presence of advanced structural heart disease in whom conventional treatments are either ineffective or technically infeasible.
- A percutaneous block can be performed at the bedside using ultrasound. Though its effects are temporary, it can be used as a bridge to a more definitive treatment such as surgical left cardiac sympathetic denervation.
- Only mild temporary side effects were seen.

## Introduction

**Fig. 1:**
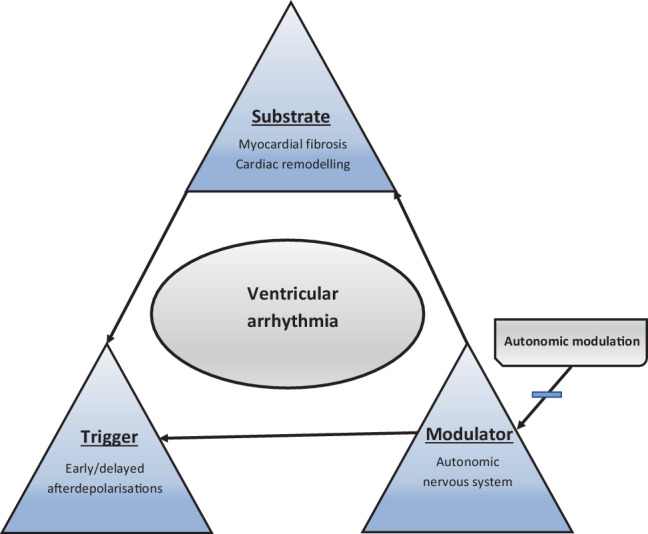
Coumel’s triangle of arrhythmogenesis provides a model for summarising the chief factors underlying ventricular arrhythmia in structural heart disease. Autonomic modulation by means of percutaneous stellate ganglion block and left cardiac sympathetic denervation aim to suppress the extrinsic sympathetic nervous system. This is suspected to be a prime modulator of arrhythmia by influencing both the trigger through altering myocardial calcium handling, which predisposes to early and later afterdepolarisations, as well as the substrate by altering conduction in the scar border zone

Ventricular arrhythmia (VA) can develop in apparently stable patients with structural heart disease due to subclinical neurohumoral hyperactivation and ventricular remodelling [1]. The most severe form of VA, electrical storm (ES), constitutes an important cause of morbidity and mortality, carrying a relative mortality risk of 3.15 [2]. Guideline-recommended first-line treatment aims to eliminate VA triggers and modulators by suppressing the cardiac sympathetic tone using a combination of beta-blockers, amiodarone and/or sedation [3]. Urgent catheter ablation (CA) and/or deep sedation are recommended when the former fail to control VA. However, CA might not be feasible for a variety of reasons, such as haemodynamic instability, substrate not amenable to ablation, ventricular thrombus or left-sided mechanical valvular prosthesis. Logistical considerations may also be a limiting factor in some centres. The temporary nature of deep sedation implies that it is primarily a bridge to more definitive treatment. The 2022 ESC guideline now classifies modulation of the autonomic nervous system (ANS) as a class IIB recommendation in selected patients with refractory VA ([3]; Fig. 1).

The pro-arrhythmogenic effects of the sympathetic branch of the ANS in structurally abnormal hearts have been described extensively [4]. Neural remodelling results in areas of sympathetic hyperinnervation and relative denervation predisposing to autonomically mediated heterogeneity of refractory periods. Cellular electrical remodelling predisposes to neurotransmitter-mediated intracellular Ca^2+^ overload and delayed afterdepolarisations. The sympathetic fibres innervating the ventricular myocardium derive from the ganglia of the cervicothoracic paravertebral chain, primarily from the left and right stellate ganglia (C7‑8 to Th1-2) [5, 6]. Surgical excision of the left stellate ganglion by means of video-assisted thoracoscopic left cardiac sympathetic denervation (LCSD) has been shown to reduce the VA burden in catecholaminergic polymorphic ventricular tachycardia and congenital long QT syndromes [7, 8]. More recently, a reduction in VA burden has also been described following percutaneous stellate ganglion block (PSGB) in patients with structural heart disease [9–15]. This percutaneous approach has the advantage of being a bedside intervention using ultrasound guidance, while major complications are rare [16]. Although its effects are temporary, expected to last no more than 2 weeks, PGSB provides a time window for scheduling definitive therapy. Since 2018, PSGB and LCSD procedures have been performed at our centre in selected patients with refractory VA.

## Methods

### Study population

This study was performed at the Medisch Spectrum Twente, Enschede, the Netherlands. Patients were identified by screening the weekly electrophysiology meetings between 1 January 2018 and 1 October 2021. Fourteen patients > 18 years of age with structural heart disease who had PSGB performed for refractory VA were identified. In two patients follow-up was incomplete, as they were transported to a transplantation centre following PSGB, so they were excluded.

### Definitions

Sustained VT was defined as having a cycle length < 600 ms with a duration of ≥ 30 s or resulting in either antitachycardia pacing (ATP) or (intracardiac) defibrillation. ES was defined as a cluster of three or more episodes of either sustained VT, ventricular fibrillation or delivery of ATP or an electrical shock by a defibrillator in ≤ 24 h [17, 18]. Structural heart disease was considered to be either a left ventricular ejection fraction of ≤ 50% or the presence of late gadolinium enhancement on cardiac MRI.

### Patient selection

Ventricular arrhythmia was treated according to the European Society of Cardiology Ventricular Arrhythmias and the Prevention of Sudden Cardiac Death guideline and the judgement of the treating physician [3]. This generally consisted of antiarrhythmic drugs, sedation and CA when possible. PSGB was considered when VA was refractory to, or not amenable to, the aforementioned treatments. Cases in which PSGB was considered were discussed at our multidisciplinary electrophysiology meeting beforehand. In the case of ES an emergency meeting was scheduled. Only when other options such as CA were not feasible, for example a known inability to approach the substrate or severe comorbidities, was PSGB considered.

### Ultrasound-guided PSGB

Percutaneous stellate ganglion block was performed by one of three anaesthesiologists with extensive experience with PSGB for chronic pain syndromes. We decided to perform a left-sided block in all cases. This decision was based on previous studies on LCSD in channelopathies as well as animal research, which suggests that the left stellate ganglion generally innervates a larger portion of the left ventricular myocardium [11, 19–23].

The procedure was performed at the bedside at the Cardiac Care Unit using an ultrasound-guided paratracheal approach. Ultrasound was used to visualise critical structures, including the carotid and vertebral arteries, the left jugular vein, the 6th cervical vertebra, the longus colli muscle and the stellate ganglion, in order to reduce the risk of major vascular complications [24]. Direct oral anticoagulants were discontinued 24 h in advance when possible. An INR of < 2.0 was desired if a vitamin‑K antagonist was administered.

While supine the patient’s head was rotated to the right and a sterile field was created around the neck. After local anaesthesia of the skin a 5-cm echogenic needle (Pajunk, Geisingen, Germany) was inserted in plane from lateral towards medial using the paratracheal approach with a route dorsal to the carotid artery and jugular vein to the stellate ganglion area. An aspiration test was performed and, if negative, the injection was performed. Initially, the type of anaesthetic agent used was determined by the operator. Subsequently, a protocol was implemented whereby 5 ml levobupivacaine 0.25% and 10 mg dexamethasone were used. Dexamethasone is co-administered to shorten the time to onset of the block and to increase the duration for up to 2 weeks [25].

### Study endpoints

The primary outcome was the number of times ATP was delivered and the number of ICD shocks or external cardioversions performed for VA in the week before and the week following PSGB. External cardioversions were counted as shocks. This period was chosen to both account for the daily variation in VA episodes as well as PSGB being used as a bridge to a more definitive treatment which would influence the arrhythmia burden. Due to limitations of the patient monitoring system and ICD therapy being disabled in several patients with ES, we were unable to accurately reconstruct the exact onset of VA on the day of PSGB in several cases, and this was therefore not reported. Charts were checked for potential complications, such as local haematoma, paravertebral haematoma, vascular damage, or neurological symptoms.

### Statistical analysis

Baseline characteristics (Tab. 1) were evaluated with a mean ± standard deviation being reported. Categorical data are presented as number/total cases (percentage). Event rates before and after PSGB were compared using the Wilcoxon signed rank test. A two-tailed probability value of < 0.05 was considered statistically significant. All statistical analyses were performed with IBM SPSS Statistics for Windows, Version 24.0 (IBM Corp., Armonk, NY, USA).

**Table 1:** Patient baseline characteristics (*n* = 12)

Age (years)	73 (± 5.8)
Male sex (%)	12 (100)
Years under cardiological treatment	19 (± 8.5)
Ischaemic cardiomyopathy (%)	9 (75)
Non-ischaemic dilated cardiomyopathy (%)	3 (25)
Left ventricular ejection fraction (%)	35 (± 12.2)
ICD of any type (%)	12 (100)
CRT (%)	5 (41.7)
VT cycle length (ms)	392 (± 88)
VT cycle length > 400 ms (number of patients) (%)	6 (50)
First block in the setting of electrical storm (%)	8 (67)
*Antiarrhythmic drugs at admission*	
– Class 1	0
– Beta-blocker (class II) (%)	8 (67)
– Sotalol (%)	3 (25)
– Amiodarone (%)	8 (67)
*Heart failure medication at admission*	
– ACE inhibitors/AT2 antagonist (%)	7 (58)
– Sacubitril/valsartan (%)	4 (33)
– Mineralocorticoid receptor antagonist (%)	6 (50)
*Anticoagulant or antiplatelet medication*	
– Aspirin (%)	3 (25)
– Dual antiplatelet therapy (%)	2 (17)
– Direct oral anticoagulant (%)	4 (33)
– Vitamin K antagonist (%)	3 (25)
*Previous VT ablation*	
– Endocardial (%)	9 (75)
– Epicardiac (%)	0
Previous cardiothoracic surgery (%)	4 (33)

*ICD* implantable cardioverter defibrillator, *CRT* cardiac resynchronisation therapy, *VT* ventricular tachycardia, *ACE* angiotensin-converting enzyme, *AT2* angiotensin II

## Results

Twelve patients were identified who underwent PSGB for refractory VA and underlying structural heart disease during the study period. Median follow-up was 601 days (± 529). All patients were men, aged between 51 and 85 years of age, with monomorphic VT (Tab. 1). Gender did not play a role in patient selection for PSGB, and autonomic modulation has been performed for various indications in both genders at our centre. Time between either initial diagnosis of a cardiomyopathy or first myocardial infarction and PSGB was a mean of 19 years (± 8.5 years). The majority of patients (75%) had ischaemic cardiomyopathy with the remainder having a non-ischaemic dilated cardiomyopathy. Median left ventricular ejection fraction was 32% (± 12%). Endocardial VT ablation had previously been performed in 9 of 12 patients (75%). In the remaining 3 patients prior VT ablation had been deferred either due to comorbidity or because important technical limitations were expected due to patient-related factors. At baseline, 8 of 12 (67%) patients were already being treated with the combination of both beta-blocker and amiodarone. Three were on sotalol, and one patient was treated with bisoprolol (Tab. 2).

**Table 2:** A description of the individual cases. All patients are male. All ventricular tachycardia (*VT*) ablations performed were endocardial procedures unless otherwise specified

Patient	Age (years)	CMP type	LVEF (%)	AAD at admission (daily dosage)	Previous VT ablation	Presentation with	VT CL (ms)	Clinical context for resorting to autonomic modulation
1	69	Ischaemic	15	Amiodarone 200 mg Carvedilol 12.5 mg	Yes	Recurrent VT	510	Patient refused to undergo repeat CA with mechanical circulatory support in the setting of recurrent haemodynamically not tolerated slow VT
2	51	Ischaemic	10	Amiodarone 400 mg Metoprolol 150 mg	No	Recurrent VT	476	CA deferred because technical difficulties were expected due to severe LV dilatation (LV end-diastolic diameter of 90 mm)
3	62	Non-ischaemic	45	Amiodarone 200 mg Bisoprolol 10 mg	No	Recurrent VT	390	Strong suspicion of an epicardiac substrate with difficult access expected due to morbid obesity (BMI 43). Subtherapeutic amiodarone levels
4	73	Ischaemic	34	Amiodarone 300 mg Metoprolol 200 mg	Yes	Recurrent VT	508	Left-sided septal substrate with both a mechanical mitral valve prosthesis and a calcified aortic valve which could not be passed during a previous CA
5	69	Ischaemic	47	Amiodarone 200 mg	Yes	Electrical storm	275	Recent unsuccessful CA: mid-myocardial septal substrate could not be reached from either the LV or the RV
6	75	Ischaemic	26	Amiodarone 400 mg Metoprolol 50 mg	Yes	Electrical storm	450	Multiple clinical VT morphologies. Recent CA with successful ablation of a mid-inferior VT. The other clinical VTs were not inducible, although four other non-clinical VTs with haemodynamic instability were induced
7	74	Ischaemic	36	Amiodarone 300 mg Metoprolol 200 mg	Yes	Electrical storm	367	Recent unsuccessful endocardial CA due to an epicardiac basal inferior substrate. Previous CABG
8	74	Ischaemic	43	Sotalol 160 mg	Yes	Electrical storm	310	Electrical storm during elective CA with immediate reinitiation of VT. Exit could not be identified periprocedurally during ongoing resuscitation
9	77	Ischaemic	42	Sotalol 240 mg	Yes	Electrical storm	306	CA deferred due to significant comorbidity (amiodarone pneumonitis, cognitive impairment, hairy cell leukaemia)
10	71	Non-ischaemic	38	Amiodarone 400 mg Metoprolol 200 mg	Yes	Recurrent VT	420	Three previous CAs of VT exits near mechanical aortic valvular prosthesis (trans-septal approach) and at the LV crux (through the great cardiac vein using both cryoablation and alcohol)
11	71	Ischaemic	31	Sotalol 160 mg	Yes	Electrical storm	360	Recent substrate ablation of an inferoposterolateral scar with uncertainty whether the substrate was endo- or epicardiac. Opted for PSGB to allow for loading with amiodarone
12	85	Non-ischaemic	20	Bisoprolol 10 mg	No	Electrical storm	280	CA deferred due to frailty and a poor functional capacity

*CMP* cardiomyopathy, *LVEF* left ventricular ejection fraction, *AAD* antiarrhythmic drugs, *CL* cycle length, *CA* catheter ablation, *BMI* body mass index, *LV* left ventricle, *RV* right ventricle, *CABG* coronary artery bypass grafting

**Fig. 2:**
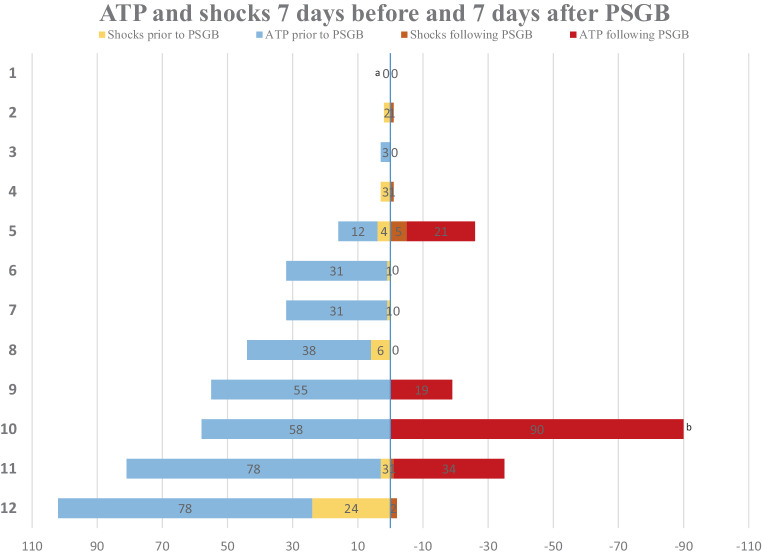
Antitachycardia pacing (*ATP*) and implantable cardioverter defibrillator (*ICD*) shocks/external cardioversions delivered in the week before and following percutaneous left stellate ganglion block (*PSGB*). ^a^Symptomatic recurrent slow ventricular tachycardia (*VT*) was not always registered by the ICD and thus the VT burden could not be reconstructed. ^b^Estimated VT burden. The exact burden could not be retrospectively reconstructed due to limitations of the inpatient recording system

After PSGB a reduction in the absolute number of ATP episodes and ICD shocks was seen in 10 of 12 patients (83%) (Fig. 2), which was significant (*p* = 0.04) In the entire cohort the decrease in ATP, although showing a clear trend, did not reach statistical significance (*p* = 0.066), whereas ICD shocks did decrease significantly (*p* = 0.028). One patient ultimately developed refractory incessant VT after an initial reduction in VA (patient 12). In the presence of advanced heart failure and severe comorbidities treatment was discontinued. In total, 4 of 12 (33%) patients died during long-term follow-up at a median of 555 days (54–1327). The causes of death were refractory VA (one case), worsening heart failure/malignancy (one case) and complicated pneumonia (two cases) (Tab. 3).

**Table 3:** A description of treatments and outcomes in the individual cases. All catheter ablations performed were endocardial. A patient was deemed either a percutaneous stellate ganglion block (*PSGB*) responder or non-responder during our follow-up multidisciplinary electrophysiology meetings

Patient	Acute treatment	Sedation	PSGB responder	Long-term treatment	Outcome
1	Urgent PSGB	No	Yes	LCSD	Infrequent VA responsive to ATP recurred 15 months after LCSD. Deceased due to malignancy and worsening heart failure
2	Lidocaine	No	No	LCSD	No follow-up data available after transferral to a cardiac transplantation centre 2 weeks after LCSD
3	Amiodarone	No	Yes	Additional i.v. amiodarone	Free from VA after therapeutic amiodarone levels were achieved
4	Lidocaine	No	No	Catheter ablation	Urgent catheter ablation of a basal septal exit was performed after VA recurrence following PSGB. Deceased due to pneumonia
5	Amiodarone	Yes	Yes	LCSD	VA recurred following LCSD. Stabilised after two catheter ablations, both with extensive substrate modification
6	Urgent PSGB	No	No	LCSD	Deceased due to refractory VA 8 weeks after LCSD
7	Urgent PSGB	No	Yes	Catheter ablation	LCSD not feasible due to adhesions. Percutaneous radiofrequency stellate ganglion block was performed. After VT recurrence 6 weeks later referred to a university hospital
8	Amiodarone	Yes	Yes	Amiodarone i.v.	Free from VA
9	Sotalol and urgent PSGB	No	Yes	LCSD	Free from VA
10	Urgent PSGB	No	Yes	LCSD	Infrequent asymptomatic VT terminated by ATP recurred 6 months after LCSD
11	Sotalol and urgent PSGB	No	Yes	Amiodarone i.v.	Free from VA. Deceased due to pneumonia 2 years after LCSD
12	Amiodarone	No	No	Amiodarone i.v.	Deceased. Treatment discontinued after VT recurrence on day 2 after PSGB

*VA* ventricular arrhythmia, *VT* ventricular tachycardia, *PSGB* percutaneous stellate ganglion block, *LCSD* left cardiac sympathetic denervation, *ATP* antitachycardia pacing

In three patients a second or even third PSGB was performed as a bridge to LCSD after VA recurred more than a week after the initial block. Two PSGBs resulted in a VT-free interval of 12 and over 14 days, allowing LCSD to be scheduled. In the third patient VT recurred at day 3 in the context of a COVID-19 infection.

Following PSGB one patient complained of temporary hoarseness. Anisocoria was reported in 2 of 12 (17%) blocks. In one patient temporary hypotension during pre-existing therapy-refractory incessant VT was seen immediately following PSGB but resolved after ‘spontaneous’ conversion to sinus rhythm 2 h later. No vascular complications were observed.

LCSD was performed in five patients following PSGB. Antiarrhythmic drugs had been unchanged in the weeks before and following surgery. One patient with cessation of VA following PSGB had a recurrence of ES 8 days after LCSD. We are unable to explain this difference in response, as his treatment had otherwise been unchanged before and following both PSGB and LCSD. In one patient with a mid-septal VT circuit not amenable to ablation LCSD did not reduce the VA burden, but higher amiodarone levels resulted in adequate VA suppression. The other three patients had substantial reduction of their VA burden with one remaining completely free of VA for 15 months, but he passed away due to a combination of disseminated bladder cancer and worsening heart failure. A second patient with 2281 instances of ATP in the 6 weeks preceding LCSD had only 1 h of recurrent VA terminated by ATP 13 days after LSCD. Only non-sustained VT was seen 6 months after LCSD. After these 6 months VT occurred only infrequently, but could be terminated by ATP. The third patient had only two episodes of VT terminated by ATP in the year following LCSD and a recurrence of ES a year later.

## Discussion

We found PSGB to be efficacious in stabilising recurrent drug-refractory VA in the majority of patients with severe structural heart disease in whom urgent CA was deemed to be either technically infeasible or to carry an unacceptably high risk. We consider the comparative simplicity of the procedure and low risk of major complications to be advantages of PGSB. Another potential benefit of PGSB is identifying patients who could benefit from LCSD for long-term suppression of VA.

There are several limitations to the data presented here. With antiarrhythmic drugs and CA being the preferred treatment for VA, there is an implicit selection bias in opting for autonomic modulation. It was not feasible to create a matched historical cohort. Thus, in the absence of a control population the reduction in VA burden might in part be attributed to a late effect of the antiarrhythmic drugs administered. However, with two-thirds of patients already receiving long-term treatment with amiodarone/beta-blocker, an antiarrhythmic effect of additional amiodarone would not be expected. The absence of females in this series is also notable. Gender was not a factor when considering patients for autonomic modulation.

We speculate that some non-responders might have benefitted from a subsequent right-sided PSGB, as animal research has demonstrated that in canines the left and right stellate ganglion innervate different segments of the myocardium. It is plausible that the sympathetic innervation pattern is similar in humans [6, 21–23]. Secondly, in non-responders, e.g. those with scar-related arrhythmias, the ANS might be only a minor modulator. Finally, ineffective blockade of the stellate ganglion might be a factor in non-responders. However, this is unlikely, as extensive spread of agents injected into the paravertebral space has been previously demonstrated [26–29]. Methods to quantify cardiac autonomic tone would certainly be helpful in differentiating between the above hypotheses. We found traditional ANS markers, such as QTc dispersion and heart rate variability, to be impracticable in our patients due to the presence of frequent ventricular ectopia and/or permanent (bi-)ventricular pacing or antiarrhythmic drugs. MIBG (metaiodobenzylguanidine) scans were also logistically infeasible in unstable patients. Further research into novel methods, such as sensory skin nerve activity, are of particular interest for evaluating the response to PSGB and potentially selecting patients who could benefit from autonomic modulation.

### Limitations

Only a limited number of patients were studied. PSGB/LCSD was performed only on male patients during the study period. No reliable data are available on the exact change in VA in the first 24 h following PSGB. Moreover, a reliable method is lacking for measuring the cardiac autonomic tone before and following PSGB in patients with refractory VA.

## Conclusion

In this retrospective series, left-sided PSGB showed significant promise as a treatment to temporarily suppress VA and ES in patients with extensive structural heart disease considered not amenable to urgent CA. Cessation of VA was seen in several patients for whom no other evidence-based acute treatment options were available, allowing definitive treatment to be scheduled. Further randomised studies are required to determine the role PSGB might play in the treatment of VA. Moreover, methods for quantifying the change in cardiac sympathetic tone, which has been achieved following a percutaneous block, are of particular interest.
